# Longitudinal Changes in the Motor Learning-Related Brain Activation Response in Presymptomatic Huntington's Disease

**DOI:** 10.1371/journal.pone.0154742

**Published:** 2016-05-18

**Authors:** Florian Holtbernd, Chris C. Tang, Andrew Feigin, Vijay Dhawan, Maria Felice Ghilardi, Jane S. Paulsen, Mark Guttman, David Eidelberg

**Affiliations:** 1 Center for Neurosciences, The Feinstein Institute for Medical Research, Manhasset, New York, United States of America; 2 Department of Neurology, Northwell Health, Manhasset, New York, United States of America; 3 Department of Physiology, Pharmacology, and Neuroscience, City University of New York Medical School, New York, New York, United States of America; 4 Department of Psychiatry, University of Iowa Carver College of Medicine, Iowa City, Iowa, United States of America; 5 Department of Neurology, University of Toronto, Toronto, Ontario, Canada; University of Ulm, GERMANY

## Abstract

Neurocognitive decline, including deficits in motor learning, occurs in the presymptomatic phase of Huntington’s disease (HD) and precedes the onset of motor symptoms. Findings from recent neuroimaging studies have linked these deficits to alterations in fronto-striatal and fronto-parietal brain networks. However, little is known about the temporal dynamics of these networks when subjects approach phenoconversion. Here, 10 subjects with presymptomatic HD were scanned with ^15^O-labeled water at baseline and again 1.5 years later while performing a motor sequence learning task and a kinematically matched control task. Spatial covariance analysis was utilized to characterize patterns of change in learning-related neural activation occurring over time in these individuals. Pattern expression was compared to corresponding values in 10 age-matched healthy control subjects. Spatial covariance analysis revealed significant longitudinal changes in the expression of a specific learning-related activation pattern characterized by increasing activity in the right orbitofrontal cortex, with concurrent reductions in the right medial prefrontal and posterior cingulate regions, the left insula, left precuneus, and left cerebellum. Changes in the expression of this pattern over time correlated with baseline measurements of disease burden and learning performance. The network changes were accompanied by modest improvement in learning performance that took place concurrently in the gene carriers. The presence of increased network activity in the setting of stable task performance is consistent with a discrete compensatory mechanism. The findings suggest that this effect is most pronounced in the late presymptomatic phase of HD, as subjects approach clinical onset.

## Introduction

The acquisition, consolidation and retrieval of serial movements are essential in everyday life and behavioral adaptation. A large body of functional imaging studies investigated the neuronal correlates of motor sequence learning in healthy subjects and individuals with brain disease [1–3]. According to a comprehensive systems-based model of this cognitive process, the acquisition and retrieval of sequential information is mediated by specific cortico-striatal, cerebello-thalamo-cortical, and cortico-cortical projection pathways. In this context, neurodegenerative disorders involving the basal ganglia can disrupt the function of learning-related pathways linking the affected regions with the cerebral cortex, leading to compromised task performance. By the same token, such changes may be accompanied by compensatory increases in task-related activity in regions unaffected pathologically by the disease process. In this vein, increases in learning-related activation and functional connectivity in cerebellar pathways have been consistently discerned in individuals with basal ganglia disorders allowing for performance to be maintained at or near normal levels [4, 5]. Indeed, we have found that compensatory responses of this type can persist for extended periods of time in subjects with early Parkinson’s disease [6].

Whether analogous compensatory changes are evident in individuals with preclinical/prodromal disease is not known at either the regional or systems level. More specifically, information is limited regarding the neural substrates underlying the development of cognitive deficits prior to clinical onset. To address these issues, we examined the changes in motor sequence learning and associated neural activation responses that took place over time in a cohort of preclinical carriers of the Huntington’s disease (pHD) mutation. The diagnosis of HD relies on the presence of overt motor signs with chorea being the main feature. Nonetheless, neurocognitive deficits appear commonly in the presymptomatic stage of the disease [7]. Indeed, alterations in fronto-striatal and fronto-parietal connectivity have been linked to these deficits [8, 9], particularly in relation to sequence learning [10]. In general, cognitive dysfunction becomes more severe as subjects approach phenoconversion [7, 11]. That said, few studies have longitudinally evaluated task-related activation responses in this population.

In this study we utilized ^15^O-labeled water (H_2_^15^O) positron emission tomography (PET) in conjunction with network analysis to identify compensatory changes in learning-related neural activation that take place with the progression of preclinical HD.

## Material and Methods

### Subjects

Ten right-handed presymptomatic HD subjects (5 men and 5 women; age 47.3±10.7 (mean±SD) years; mean CAG repeat length 41.6±1.8) were recruited through the Movement Disorders Center of the Northwell Health. The predicted number of years to clinical onset (YTO) was estimated for each subject based on baseline CAG repeat length and age [12]. The average time to predicted onset for the whole group was 10.6±9.0 years. Two of the subjects phenoconverted during the follow-up period (see below). For those subjects, we used the actual number of years elapsed from baseline to the time of clinical diagnosis.

All subjects were assessed clinically at baseline and follow-up (interval: 1.5±0.1 years) by a neurologist experienced in the assessment and diagnosis of pHD subjects (A.F.). At baseline, none of the subjects exhibited sufficient signs and symptoms for a clinical diagnosis of HD to be made. Nonetheless, two of the subjects were diagnosed at the 18-month follow-up time point. United Huntington’s Disease Rating Scale (UHDRS) motor scores did not differ over time for the subjects (8.3±10.5 vs. 11.3±11.6; p = 0.137, Wilcoxon signed-rank test). In this study, the pHD data were compared to baseline measurements from 10 right-handed healthy control (HC) subjects (6 men and 4 women; age 46.8±13.3 years). Baseline activation data from the pHD subjects have been reported previously [10].

### Behavioral tasks

All subjects performed two motor tasks during each session of PET scanning: a motor sequence learning task (**LEARN**) and a kinemetically equivalent motor execution task (**MOVE**) [13]. Briefly, in both tasks, the dominant right hand was used to move a cursor on a digitized tablet. Out and back reaching movements were executed from the central position toward one of eight radial targets displayed on the screen. In **LEARN**, the eight targets appeared in pseudo-randomized order without repeating elements at a 1s tone interval. Subjects were asked to learn the presented target sequence and to reach the targets in synchrony with the tone. The sum of all correctly anticipated targets in each **LEARN** run was computed and defined as the retrieval index (RI). Thus, a high RI reflects good learning performance. In **MOVE**, a kinematically controlled motor execution reference task, the targets appeared in a predictable counterclockwise order at 1s intervals matched to a cueing tone. Subjects were instructed to reach the targets in synchrony with the tone such that the movement had to be initiated before the target appeared. In addition, subjects were scanned in a non-movement, non-learning resting (**REST**) condition, in which they were instructed to watch the targets without moving or attempting to learn a sequence. The subjects were trained outside the scanner one day prior to the scan to ensure stable task performance during the imaging epoch.

### Positron emission tomography

All pHD subjects underwent H_2_^15^O PET at baseline and follow-up; the healthy control subjects were scanned only at baseline. Subjects fasted for a minimum of 6 hours before imaging. Scanning was conducted using the GE Advance tomograph (GE Healthcare) in 3D mode as described previously [10], while the subjects performed the **LEARN**, **MOVE**, and **REST** tasks in randomized order. The pHD subjects performed the **MOVE** and **LEARN** tasks twice at both baseline and follow-up. Thus, 20 **LEARN**/**MOVE** scan pairs were available for analysis at each of the two longitudinal pHD time points. However, because of fatigue, one of the healthy subjects performed only a single trial of each task, which resulted in 19 **LEARN**/**MOVE** scan pairs for the control group. All but one of the pHD subjects were scanned twice at each time point in the **REST** condition. Eight of the 10 control subjects were scanned twice in the **REST** condition; the remaining two subjects were scanned only once in this condition.

Relative regional cerebral blood flow (rCBF) was estimated using a modified slow bolus method [14], in which up to 12 mCi of H_2_^15^O in 4 mL saline was injected by an automatic pump in 16 sec (15 mL/min) followed by a manual 3 mL saline flush. There was a time delay of approximately 17 sec in arrival of radioactivity in the brain, and the time from rise to peak count rate was 35–40 sec. The timing of task initiation was individually adjusted so that the arrival of radioactivity occurred approximately 10 sec after the start of the task. PET data acquisition began at the time of radioactivity arrival in the brain and continued for 80 sec. The end of task thus coincided with the end of data acquisition. The interval between successive H_2_^15^O administrations was 12 min to allow for the decay of radioactivity. Two H_2_^15^O boluses were injected in each task condition with a maximum of 16 boluses per study for each subject. The total effective dose equivalent of 192 mCi of H_2_^15^O was 0.81 rads or 0.0081 Gy. The maximal exposure was 0.63 rads (0.0063 Gy) for reproductive organs and 1.57 rads (0.0157 Gy) for critical organs, which were substantially lower than the limit (5.0 rads or 0.05 Gy) of dose exposure per study for each subject.

Ethical permission for the PET studies was obtained from the Institutional Review Board of Northwell Health (Manhasset, NY). Written consent was obtained from each subject after detailed explanation of the procedures.

### Data analysis

#### Preprocessing

Imaging data was preprocessed using SPM 5 (Wellcome Department of Cognitive Neurology, London, UK) implemented in MATLAB (Mathworks, Sherborn, MA). All scans from each pHD subject were spatially realigned across conditions (**REST**, **MOVE** and **LEARN**) and time points to remove possible mismatch due to subject motion between imaging sessions and repositioning errors in repeat studies. All images were averaged after the spatial realignment to produce a mean image with higher signal-to-noise ratio. Realigned images were then spatially normalized to the standard SPM PET template via the mean image. The resulting images were smoothed by a 10 mm Gaussian filter in 3D space.

At each time point, **LEARN**-**MOVE** subtraction images were constructed using an in-house program (ScAnVP 5.9.1, available at www.feinsteinneuroscience.org) to map learning-specific activation responses on a voxel-by-voxel basis. Before subtraction, images were masked by applying a whole brain gray-matter mask; an adjustment for the global mean was performed for each image. Since the pHD subjects performed two runs of each task, 20 (10×2) subtraction images were constructed for the baseline and follow-up sessions. In this way, we assessed the longitudinal changes in brain activation in the pHD subjects that were specifically associated with learning, as opposed to less specific motor execution effects.

#### Network analysis

To identify significant spatial covariance pattern(s) associated with compensatory network-level responses during sequence learning, we sought: (1) topographies with longitudinal increases in task-specific (**LEARN**-**MOVE**) activation responses over time in the H_2_^15^O PET data; and (2) patterns in which subject expression increased in proportion to baseline disease burden. Thus, we used Ordinal Trends Canonical Variates Analysis (OrT/CVA, software available at https://www.nitrc.org/projects/gcva_pca) [13, 15], a form of supervised principal component analysis (PCA), to isolate potential compensatory topographies in the pHD subjects. Specifically, we sought covariance patterns with increasing expression over time (i.e., TP2 ≥ TP1), such that subject scores were greater for the mutation carriers who were nearer to phenoconversion. In other words, we endeavored to identify activation networks for which compensatory responses were largest in individuals with greatest underlying disease burden.

To this end, we used OrT/CVA to identify a set of one or more linearly independent (orthogonal) spatial covariance patterns displaying monotonic changes in expression across conditions or time points. In contrast to univariate analytic approaches comparing group means, this multivariate approach identifies ordinal trends in the data, i.e., monotonic increases or decreases in pattern expression across conditions/time points with few if any individual case violations. Once a significant spatial covariance pattern is identified, subject scores, representing network expression in individual cases, are computed for scans acquired at the various time points or experimental conditions [13, 16]. The significance of subject score difference between groups/conditions is determined using non-parametric permutation tests. The reliability of the voxel weights (regional loadings) on the identified network topography is determined by bootstrap resampling procedures [17].

In the current study, OrT/CVA was applied to subtraction images (**LEARN**-**MOVE**) from the pHD subjects obtained at baseline and at the 18-month follow-up time point. In a second step, subject scores for the resulting principal component (PC) patterns were entered into a multiple regression model using baseline YTO as a measure of disease burden for each individual. Compensatory learning-specific activation topographies were defined as those with significantly increasing subject scores over time, for which individual differences in expression correlated positively with baseline YTO values. Model selection was based on the number of PCs associated with the smallest value for the Akaike Information Criterion [18].

For pattern validation, the subtraction images were permuted across time points to generate a null-hypothesis for the r^2^-value of the YTO-fit. Significance was assumed for p<0.05 (1,000 iterations). The YTO-fitted OrT/CVA pattern topography was displayed at a voxel-weight threshold of *z* = 2.33 (p<0.01, cluster cutoff = 100 voxel). Only voxels found to be reliable on bootstrap resampling (magnitude of the inverse coefficient of variation (|ICV|) > 1.96, p<0.05; 1,000 iterations) were considered as contributing network regions.

After network identification, pattern expression was prospectively computed in the **MOVE** and **LEARN** scans of the pHD subjects and controls using an automated voxel-based procedure [19]. Subject scores computed in the **MOVE** scans of the healthy control subjects were used to *z*-transform the **LEARN** and **MOVE** values for the pHD and control subjects, such that the mean of the control **MOVE** scans was zero with a standard deviation of one. After standardization, subject scores of the **MOVE** scans were subtracted from the corresponding scores in the **LEARN** condition. As described above, the resulting difference score reflects the learning-related network activation response. In a second step, the longitudinal change of this response between baseline and follow-up in the pHD group was computed ([**LEARN**-**MOVE**]_TP2_-[**LEARN**-**MOVE**]_TP1_) and correlated with baseline YTO values.

Lastly, to evaluate potential longitudinal changes in network expression occurring independent of task condition, we computed corresponding subject scores in the **REST** scans obtained from the pHD and healthy control subjects. As above, expression values computed in the control subjects were used to standardize the corresponding measures computed in the pHD subjects at the two time points. Longitudinal changes in resting network expression were calculated by subtracting the *z*-scores of the baseline scans from those measured at follow-up (**REST**_TP2_-**REST**_TP1_). For subjects with more than one scan per condition, the corresponding network scores were averaged before further analysis.

#### Regional analysis

For post-hoc analysis, longitudinal changes of rCBF were evaluated by centering a 5 mm sphere at the peak voxel of each network region. Regional values were measured in each region in the **LEARN** and **MOVE** scans of the pHD subjects at the two time points, and in the healthy subjects at baseline. rCBF values were adjusted for the global mean of each scan to account for potential alterations across scans. The learning-related rCBF response (**LEARN**-**MOVE**) was calculated for each scan pair. As in the network analysis, the longitudinal change in the learning-related brain activation response ([**LEARN**-**MOVE**]_TP2_-[**LEARN**-**MOVE**]_TP1_) was computed in the pHD subjects and correlated with baseline YTO values. Network regions displaying a positive correlation between learning-related activation responses and YTO corresponded to positive loadings (“red” regions) on the identified covariance pattern; those with negative correlations corresponded to negative loadings (“blue” regions) on the same pattern.

#### Statistical analysis

SPSS software was used for statistical tests. Non-parametric Wilcoxon signed-rank tests and Mann-Whitney U-tests were utilized for group comparisons where appropriate. Correlations of subject scores and rCBF values with YTO and behavioral measures were computed using non-parametric Spearman’s rank correlations. Results were considered significant for p<0.05.

## Results

### Behavioral measures

**Fig 1:**
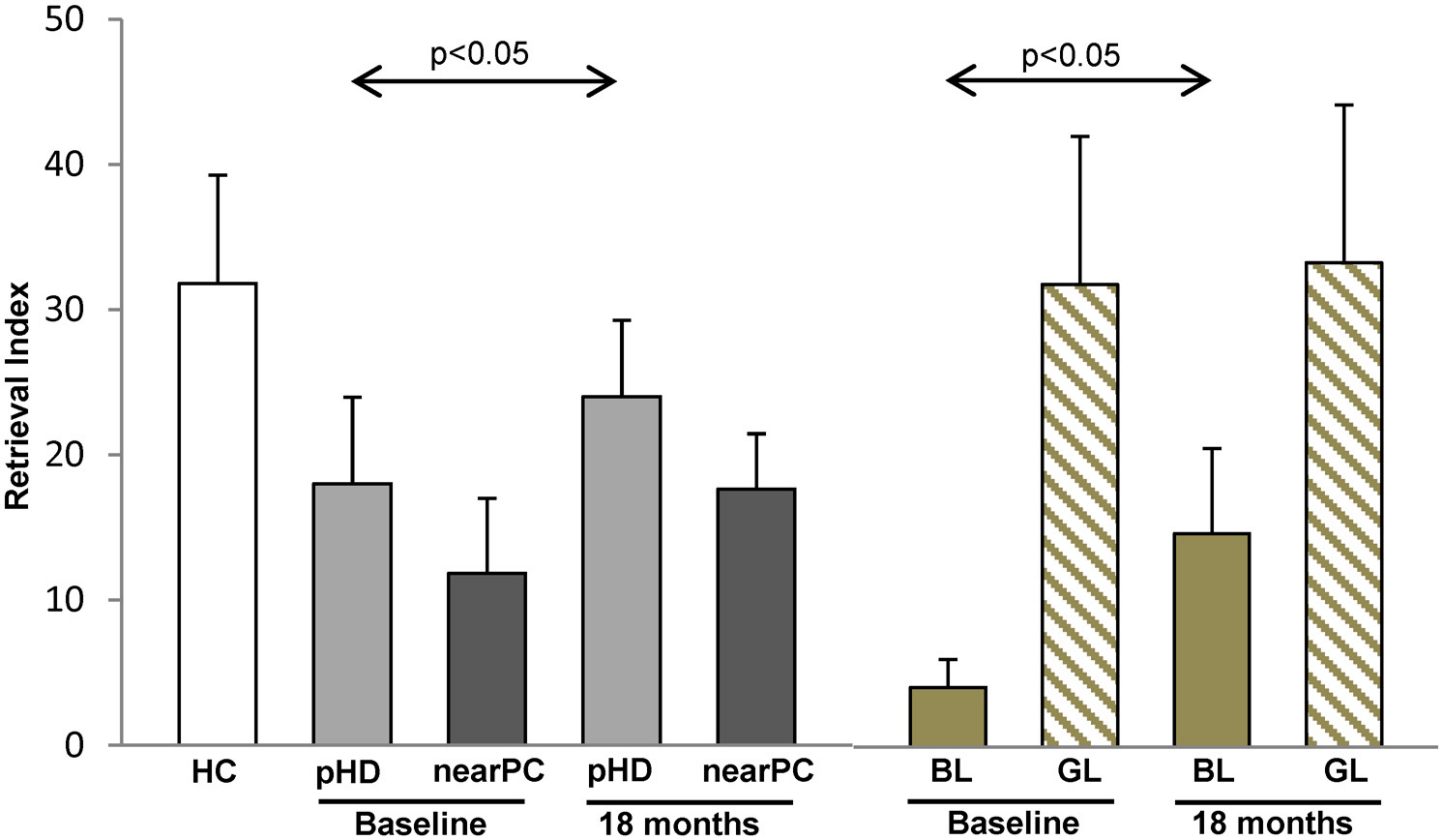
Motor learning performance in patients with presymptomatic Huntington’s disease (pHD) and healthy controls (HC). pHD subjects showed a slight improvement in learning performance at follow-up (18 months). Note that individuals with low learning performance at baseline (“bad learners,” BL) showed substantial improvement at follow-up. In contrast, “good learners” (GL) showed normal learning performance at baseline and did not change over time. Subjects with relatively high disease burden (nearPC) showed slightly lower learning performance at baseline compared to the overall group. [Bars represent group mean values. Error bars indicate SEM.]

Measures of learning performance and corresponding changes over time are displayed in Fig 1 and Table 1. No significant differences in learning performance, as measured by the RI, was present at baseline between HD mutation carriers and the healthy control subjects (pHD: 18.0±18.9; HC: 31.8±24.7, p = 0.307; Mann-Whitney U-test). Network analysis (see below) revealed that the pHD cohort was comprised of two discrete groups based upon proximity to phenoconversion (see below). Subjects in the first group were chronologically near phenoconversion (nearPC (n = 7): age 50.3±8.6 years; YTO≤11); the remaining subjects were far from phenoconversion (farPC (n = 3): age 43.3±13.6 years; YTO>11). Subjects in the nearPC group exhibited lower baseline learning performance than the cohort as a whole. Even so, mean RI in this group did not differ significantly from control values (nearPC: 11.9±13.6; HC: 31.8±24.7, p = 0.130). Borderline improvement in learning performance was seen over time for the entire pHD cohort (Delta RI (TP2-TP1): 6.0±9.0, p = 0.047; Wilcoxon signed-rank test). A similar, albeit non-significant trend was observed in the nearPC group (Delta RI (TP2-TP1): 5.8±10.8, p = 0.128). Of note, learning performance did not differ from normal at the second time point in either of the two pHD groups (p>0.49, compared to HC).

**Table 1:** Longitudinal changes in learning performance and network expression.

	RETRIEVAL INDEX			NETWORK EXPRESSION		
	TP1	TP2	Delta	TP1	TP2	Delta
**pHD**	18.0±18.9	24.0±16.6	6.0±9.0^a^	-0.7±1.1	0.5±0.8	1.3±1.9
**nearPC**	11.9±13.6	17.6±10.0	5.8±10.8	-1.3±0.6^b^	0.9±0.7^b^	2.2±1.2^a^
**BL**	4.1±2.1	14.7±7.3	10.6±7.1^a^	-1.5±0.6^b^	1.2±0.4^b^	2.8±0.9^a^
**GL**	31.9±17.7	33.3±18.8	1.4±8.9	0.1±0.8	-0.2±0.4	-0.3±1.1
**HC**	31.8±24.7	n/a	n/a	-0.1±0.4	n/a	n/a

pHD = presymptomatic Huntington’s disease, nearPC = pHD subjects approaching phenoconversion (≤11 years to predicted motor onset), BL = “bad learners” showing poor learning performance at baseline, GL = “good learners” showing good learning performance at baseline (see text for details), HC = healthy controls.

^a^p<0.05, TP1 compared to TP2.

^b^p<0.005, compared to HC.

We additionally related the longitudinal changes in network activity to baseline learning performance. Using the RI median value of 8.8 for the pHD group, five gene carriers were classified as “good learners” (GL) and five as “bad learners” (BL) based on their baseline RI values. Whereas, at baseline, learning performance was intact in the GL subjects (RI: 31.9±17.7, p = 0.902; Mann-Whitney U-test), the BL subjects exhibited a marginal reduction in this measure (RI: 4.1±2.1, p = 0.075). Interestingly, learning performance improved over time in the BL group (Delta RI (TP2-TP1): 10.6±7.1, p = 0.043; Wilcoxon signed-rank sum test), but no concurrent change was evident in the GL subjects (Delta RI (TP2-TP1): 1.4±8.9, p = 0.500). Learning performance at TP2 did not differ from normal in either group (p>0.46). Notably, the BL group was comprised of the five pHD subjects who were nearest to phenoconversion (YTO: 3.3±2.3, range 1.4–7.0). By contrast, the GL group was comprised of individuals far from the expected time of phenoconversion (YTO: 17.8±6.8, range 11.0–25.0).

### Network analysis

**Fig 2:**
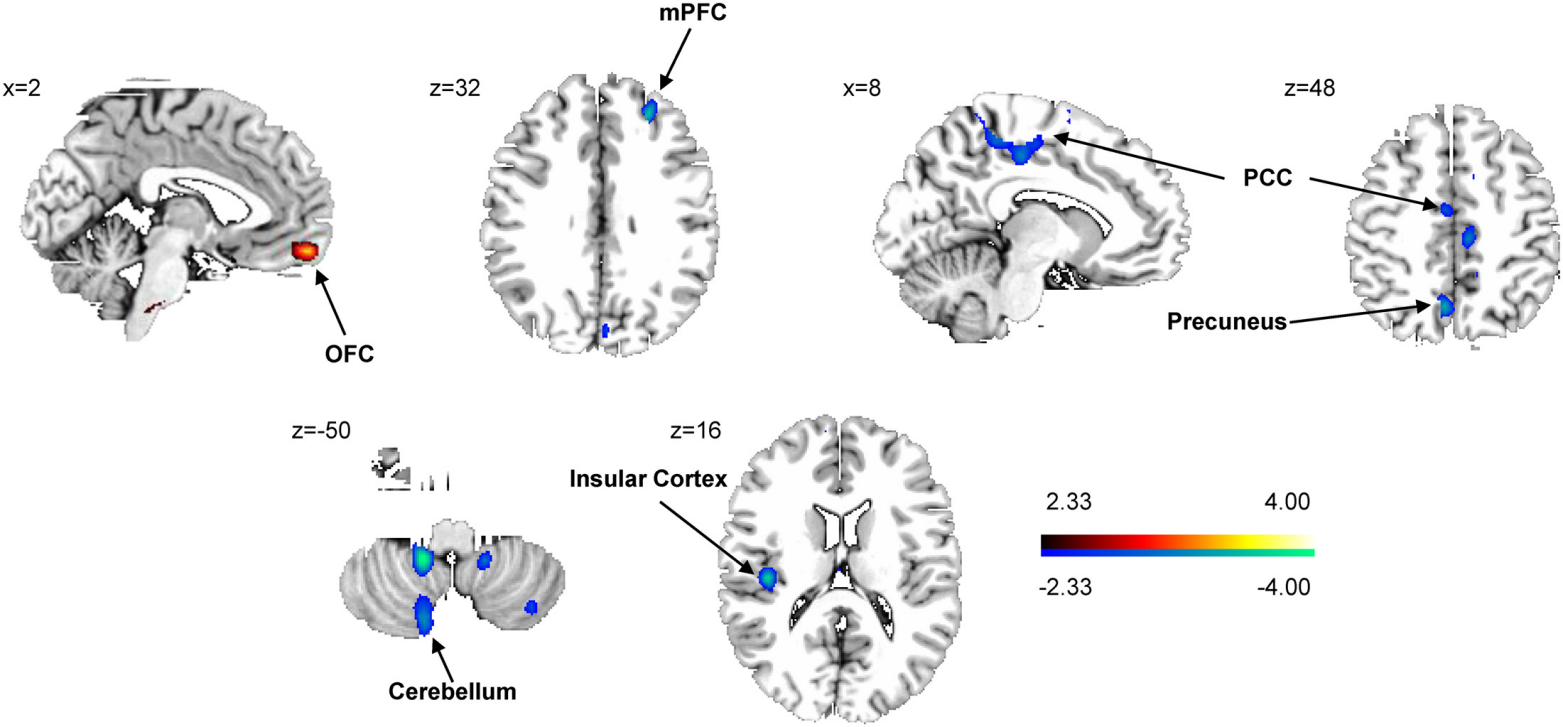
Motor learning-related spatial covariance pattern in subjects with presymptomatic Huntington’s disease. This pattern was characterized by increasing activity in the right orbitofrontal cortex (OFC; BA11) and concurrent relative decreases in the right medial prefrontal cortex (mPFC; BA10), left insular cortex (BA20), left precuneus (BA7), left cerebellum and the right posterior cingulate cortex (PCC; BA31). [The “red” blob indicates increasing activity, “blue” blobs show decreasing activity. The colored bar displays the z-score range; coordinates are displayed in MNI standard space.]

Network analysis revealed a significant pattern of learning-related activation characterized by monotonic increases in subject expression over time that correlated with baseline YTO values. This spatial covariance pattern (Fig 2) was characterized by increased learning-related activation in the right orbitofrontal cortex (BA11), associated with reductions in the right medial prefrontal cortex (BA10) and posterior cingulate (BA31) region, and in the left precuneus (BA7), insula (BA13), and cerebellum (Lob VIIA,VIIIA/B, IX). Voxel weights in these regions were found to be reliable by bootstrap estimation (|ICV| >2.81; p<0.005; 1,000 iterations), and validity of subject scores was confirmed by permutation test (p<0.001).

**Fig 3:**
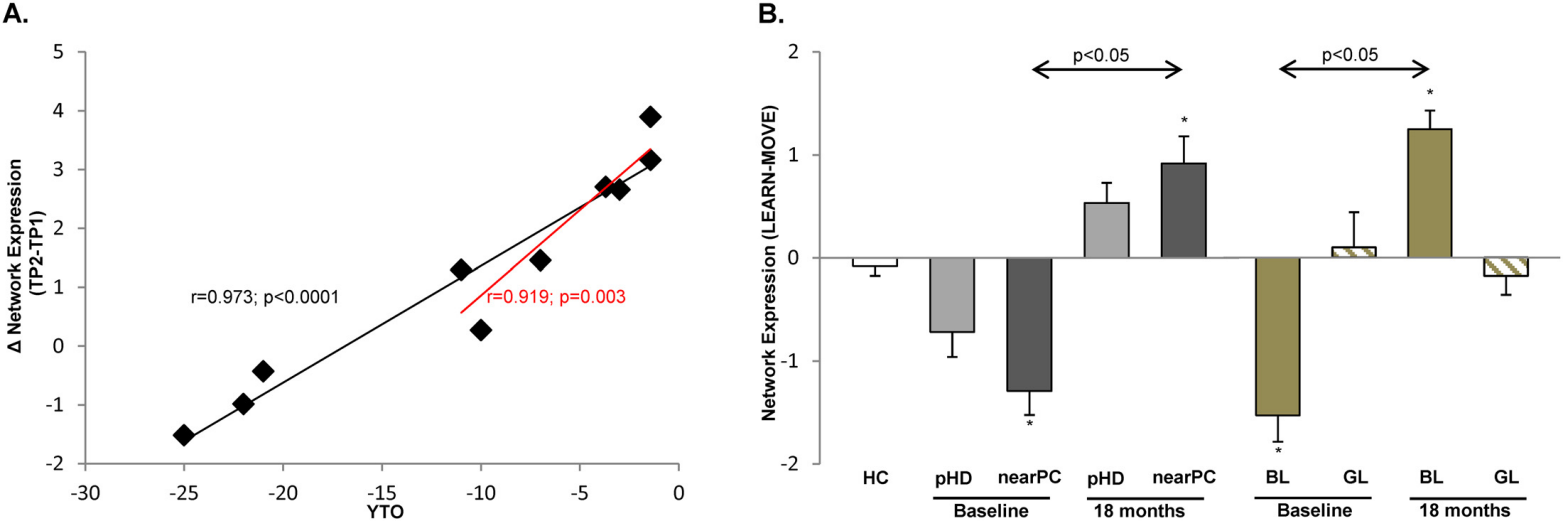
Longitudinal changes in network expression are associated with disease burden and learning performance at baseline. (**A**) Longitudinal changes in network expression (**LEARN-MOVE**)_TP2_-(**LEARN-MOVE**)_TP1_ (see text) correlated with baseline disease burden as expressed by the predicted years to disease onset (YTO). Note that the three subjects far from predicted motor onset exhibited relatively stable network expression over time, whereas subjects approaching phenoconversion (nearPC) showed a significant increase in network activity. Of note, the association of increased network expression and YTO remained significant in nearPC subjects only (*red trend line*). (**B**) Learning related brain activation (**LEARN-MOVE**, see text for details) increased significantly in nearPC subjects and individuals exhibiting poor learning performance at baseline (“bad learners,” BL). In contrast, individuals with normal learning performance (“good learners,” GL) did not show changes in network expression over time. Baseline network expression in nearPC and BL was significantly lower compared to healthy controls (HC), whereas subject scores exceeded the normal range at follow-up in these individuals. [*p<0.005, compared to HC. Bars represent group mean values. Error bars indicate SEM.]

In keeping with the proposed compensatory role of this network in pHD subjects, changes in pattern expression over time (Fig 3A) correlated with baseline disease burden (r = 0.973, p<0.0001; Spearman correlation). Thus, the most prominent longitudinal increases in network activity were observed in the pHD subjects who were nearest phenoconversion. Indeed, the nearPC subjects displayed substantial increases in network expression (mean change = +2.2 *z*-scale units) over time, whereas the farPC subjects exhibited small declines (|*z*|<1.5) in activity over the same time period. Even so, the correlation between the network changes and baseline YTO remained significant (r = 0.919, p = 0.003) when the analysis was limited to the nearPC group.

Further analysis revealed a marginal increase in network activity (p = 0.093) over time (Fig 3B; Table 1) for the overall pHD cohort. This result remained significant (p = 0.018) when only nearPC subjects were included in the analysis. Network activity for the overall group did not differ from control values at either time point (p>0.17). Nonetheless, in the nearPC group, network activity was abnormally reduced at baseline (p = 0.002). By contrast, network activity at the second time point was elevated above normal (p = 0.001) in these subjects.

These findings were similar when the pHD subjects were grouped according to their baseline learning performance. Thus, the BL subjects exhibited low network expression at baseline (p = 0.003, relative to HC), which increased significantly over time (p = 0.043) reaching super-normal levels (p = 0.002, relative to HC) at follow-up. By contrast, network expression in the GL subjects did not differ from normal at either time point (p>0.17) and did not change significantly over time (p = 0.500). Indeed, change in network expression over time was significantly greater in BL compared to GL subjects (group × time interaction effect: F_(1,8)_ = 23.234, p = 0.001; RMANOVA). No correlation was present (p>0.72) between increases in network expression over time and concurrent changes in learning performance in the pHD cohort. Of note, network expression levels measured in the resting state did not change over time in any of the pHD groups (p>0.13), and did not differ from healthy control values at either time point (p>0.17). Likewise, longitudinal changes in network activity measured in the resting condition did not correlate with baseline measures of disease burden (p>0.75) or learning performance (p>0.17).

### Regional analysis

**Fig 4:**
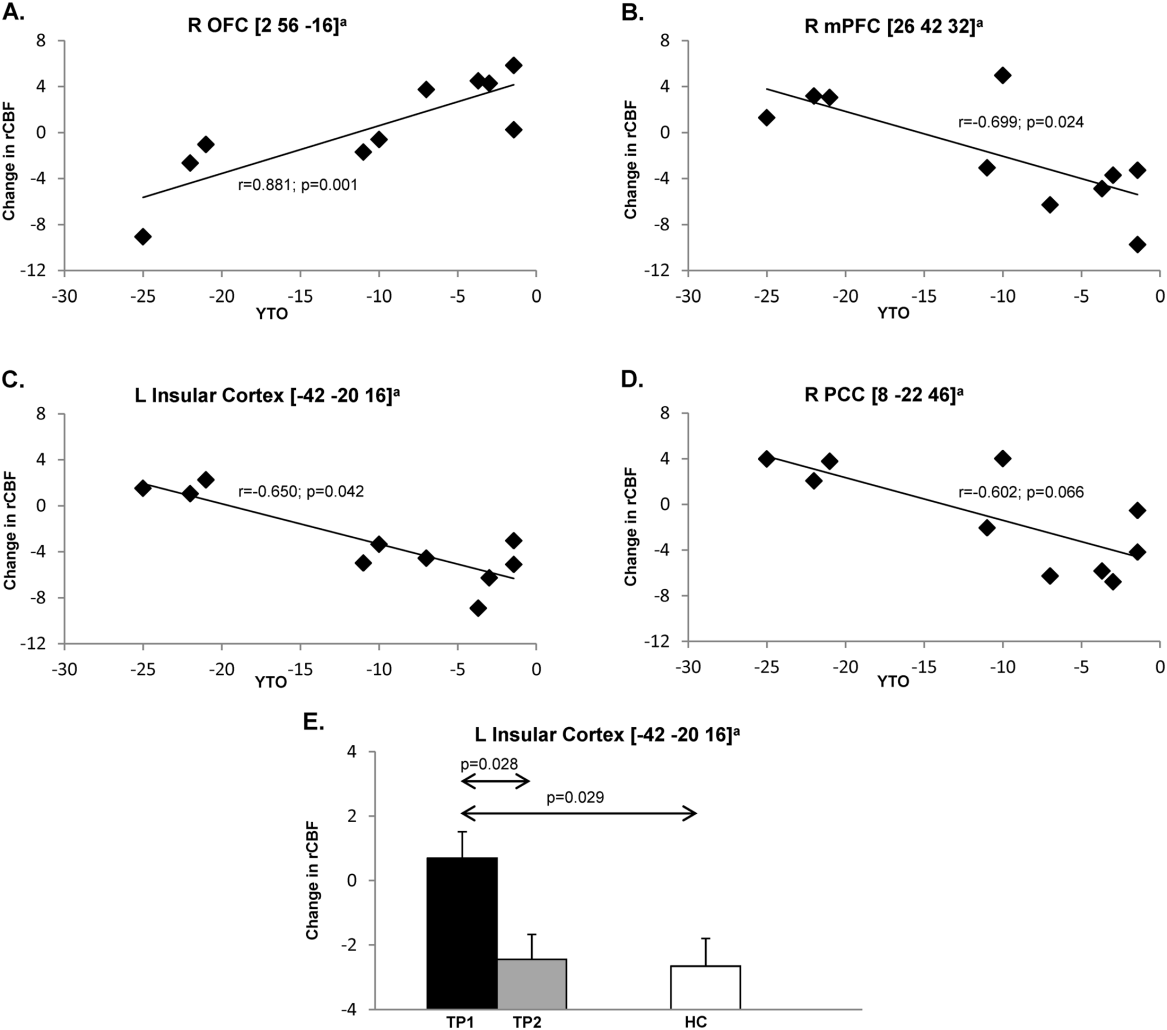
Regional correlations of learning-related brain activation changes with disease burden at baseline. At the region level, significant correlations between learning-related regional cerebral blood flow (rCBF) responses and baseline disease burden (YTO) were observed in the right orbitofrontal cortex (OFC, BA11, **A**), right medial prefrontal cortex (mPFC, BA10, **B**), and left insular cortex (BA20, **C**). Trend level significance was reached in the right posterior cingulate cortex (PCC, BA31, **D**). rCBF responses in the insular cortex exceeded that observed in healthy controls (HC) at baseline (TP1), and returned to normal at follow-up (TP2) (**E**). [Bars represent group mean values. Error bars indicate SEM. ^a^Coordinates are displayed in MNI standard space.]

The results of regional analysis are summarized in Table 2. Learning-related rCBF activation responses in the right orbitofrontal cortex (Fig 4A) correlated directly (r = 0.881, p = 0.001) with YTO values, whereas significant inverse correlations (Fig 4B and 4C) were present in the right medial frontal region (r = -0.699, p = 0.024) and in the left insula (r = -0.650; p = 0.042). A trend-level relationship (Fig 4D) between these variables (r = -0.602; p = 0.066) was noted in the right posterior cingulate cortex. Of the network regions, significant longitudinal changes in learning-related rCBF were present in the left insula (Fig 4E), in which local activation responses declined over time (p = 0.028). Compared to the healthy control subjects, baseline rCBF in this region was abnormally elevated in the pHD subjects (p = 0.029), declining toward normal by the second time point (p = 0.971, relative to HC). Similarly, a marginal decline in learning-related rCBF was also observed in the right precuneus (p = 0.047). However, rCBF at this node did not differ from normal at either time point (p>0.12). Changes in learning-related rCBF over time were not significant (p>0.20) for the other network regions. Changes in learning-related activity recorded over time in the network regions did not correlate significantly (p>0.07) with concurrent changes in task performance.

**Table 2:** Regional activity changes at the network nodes.

	NETWORK NODES				rCBF				*r*^d^	*p*
	MNI	BA	Size	T_max_	HC	pHD TP1	pHD TP2	pHD TP2-TP1		
***Positive***										
R OFC	2 56–16	11	117	3.51	-2.3±3.4	-2.3±3.0	-2.0±3.3	0.4±5.4	*0*.*881*	*0*.*001*
***Negative***										
L Insula	-42–20 16	20	148	3.75	-2.7±3.3	0.7±2.7^b^	-2.4±2.3	-3.1±3.7^c^	*-0*.*650*	*0*.*042*
R mPFC	26 42 32	10	208	3.64	-0.1±1.8	2.2±3.9	0.3±2.8	-1.8±4.8	-0.699	0.024
R PCC	8–22 46	31	278	3.17	-1.9±4.1	-0.1±4.1	-1.3±2.1	-1.2±4.4	*-0*.*602*	*0*.*066*
L Prec	-6–62 48	7	170	3.49	1.4±3.1	3.9±3.0	1.0±2.2	-2.9±4.1^c^	*-0*.*416*	*0*.*232*
L Cer	-14–46–50	IX	297	4.04	1.3±1.5	0.0±4.4	-2.8±3.6	-2.8±6.4	*-0*.*540*	*0*.*107*
		VIIA/B								
		VIIA^a^								

OFC = orbitofrontal cortex, Insula = insular cortex, mPFC = medial prefrontal cortex, PCC = posterior cingulate cortex, Prec = precuneus, Cer = cerebellum, rCBF = learning-related (**MOVE-LEARN**) change in normalized regional cerebral blood flow at each time point (TP) and for healthy controls (HC), pHD TP2-TP1 = longitudinal change in the learning related activation response (see text for details). Values are presented as mean±SD.

^a^Lobules according to the atlas of Schmahmann [42].

^b^p<0.05, compared to HC.

^c^p<0.05, TP1 compared to TP2.

^d^Correlation of pHD TP2-TP1 with baseline years-to-onset.

## Discussion

We identified a learning-related functional brain network defined by longitudinally covarying changes in the activation responses in presymptomatic HD subjects. Cognitive impairment including deficient motor learning performance often precedes the onset of motor symptoms in HD mutation carriers [10, 20]. Indeed, altered cognitive function can emerge decades before the predicted time of diagnosis, and changes in regional brain function can antecede these behavioral changes [21]. That said, recent data suggest that cognitive decline and concurrent brain changes are most prominent in the decade preceding clinical diagnosis [7, 22–24]. Moreover, brain atrophy and functional decline progress faster in gene carriers with higher disease burden [22, 24, 25]. In line with these findings, we observed pronounced elevations in network activity in pHD individuals approaching predicted motor onset.

This increase in network activation was accompanied by concurrent improvement in learning performance. Although we did not observe a significant correlation of network scores and retrieval index, this association suggests that the network compensates to some degree for the decline in learning performance expected based upon disease progression. Thus, the greatest longitudinal increases in network expression were observed in the mutation carriers with the largest disease burden and the lowest learning capacity at baseline. Indeed, significant reductions in network expression were initially present in these individuals, reaching abnormally elevated levels by the time of follow-up. By contrast, pHD subjects with normal learning performance and low disease burden did not activate this compensatory network. The specificity of the network changes for motor learning is underscored by the observation that expression values for the same covariance pattern, computed in resting (non-movement, non-learning) scans from the same subjects/time points, did not differ from normal, change over time, or correlate with baseline disease burden estimates. Taken together these findings suggest that the current network is specific for longitudinal learning-related changes in pHD, independent of overall disease progression. Indeed, this task-specific HD network did not correlate (r = 0.025, p = 0.403) with the general metabolic progression spatial covariance topography that was recently characterized in this population [26].

Unexpectedly, we observed a slight but significant improvement of learning performance in pHD subjects. Although the scans were 1.5 years apart, we cannot exclude a practice effect. Indeed, it has been reported previously that such a training effect can lead to significant improvement of cognitive performance in pHD subjects with relatively low disease burden [27]. In our cohort, the improvement in learning performance was greatest in the pHD subject with initially poor learning performance. These individuals also had low network expression at baseline and exhibited large increases in network activity over time. Thus, the data suggest that cognitive performance has to fall below a certain threshold to activate the compensatory brain network. In addition, these observations suggest that practice/training may produce improvement in learning in pHD even in individuals close to disease onset.

The learning-related topography included several regions that have been previously associated with motor learning in healthy subjects [2, 28–30]. In the right orbitofrontal cortex, learning-related rCBF values increased over time and significantly correlated with baseline disease burden. By analogy to changes seen at the network level, increasing activity in this region, which is not typically deployed for motor learning, served a compensatory role. Along these lines, we have noted an abnormal increase in task-related activation in a similar area in the baseline scans of this pHD cohort [10]. That said, the other major network regions showed an inverse correlation with baseline disease burden and tended to exhibit lower learning-related activity at follow-up than at baseline. Disease progression is a plausible explanation for decreasing activity in these brain areas. We note that these regions are activated during learning in healthy subjects, with higher rCBF during the performance of **LEARN** relative to **MOVE** [28, 31]. The decline in learning-related activation observed in these regions may reflect the incipient development of HD pathology. Indeed, longitudinal MRI data from these subjects have revealed ongoing volume loss in a number of relevant brain regions [26].

Moreover, prior structural MRI studies have consistently found volume loss in multiple brain regions in presymptomatic and early manifest HD. Loss of striatal volume [22, 32] has been associated with deficits in motor performance [33]. Loss of cortical gray matter volume or cortical thinning has been found to be most pronounced in the sensorimotor and occipital regions [34, 35] and has been linked to deficits in motor and cognitive function [33, 36]. While decreased striatal volume can be observed in gene carriers far from predicted onset of motor symptoms [22, 32], loss of cortical volume seems to be pronounced in premanifest subjects close to phenoconversion [37]. The frontal and temporal areas, however, seem to be largely spared from atrophy in premanifest HD [35, 37]. In the present study, we observed reduced learning-related activity in the posterior cingulate cortex, precuneus, and the cerebellum, regions in which volume loss has been found in premanifest and early manifest HD subjects [35, 38, 39]. In contrast, the orbitofrontal cortex, a major network node exhibiting increased activity over time, has been found to be spared from degenerative processes in presymptomatic gene carriers and is also not typically involved in motor learning [10, 35]. Other network nodes, i.e., the medial frontal cortex and insular cortex, are likewise not typically affected by brain atrophy in premanifest HD [35]. Together, these findings suggest that motor learning-related network activity, though partially linked to concurrent regional volume loss, is unlikely to be driven entirely by brain atrophy.

We acknowledge some limitations of our study. Firstly, the sample size was relatively small, and the results therefore have to be interpreted with caution. Because of the small sample size and high variability of VOI data, no correction for multiple comparisons was applied to the analyses of regional activity that were limited to the major nodes of the rigorously validated learning network. That said, the observations are valuable given that to date very few studies have focused on the longitudinal changes of brain activation that take place in pHD subjects during cognitive effort [40]. Secondly, the normal volunteers were assessed only at a single time point. Consequently, we could not directly compare changes in network expression that took place over time in the two groups. However, it is not likely that learning-related brain activity changes substantially over an interval of 18 months in healthy, young individuals. Indeed, in a recent fMRI study, no change in brain activation was present in healthy subjects repeating an attention task after two years [40]. However, we cannot fully exclude possible test-retest effects or unspecific activation effects that might have confounded network identification. Nevertheless, by implementing a non-learning control task in the study design, the risk of such effects is substantially diminished as brain activation caused by movement is the main confounder of all motor learning paradigms. Thirdly, we chose to use H_2_^15^O PET to study brain activity because it is a direct measure of rCBF with a better signal-to-noise ratio compared to functional MRI [41]. Nonetheless, due to the small sample size and the exploratory nature of this study, it might be worthwhile to further examine and validate the current findings in future functional or perfusion MRI studies with larger samples.

Finally, we did not observe a linear relationship between learning scores and the brain changes that occurred at either the region or network levels. Even so, the simultaneous occurrence of increasing network activity and improved motor learning is suggestive of such an association, particularly given the absence of corresponding changes in the resting state. In this vein, it is conceivable that individual differences in task performance are mediated by a separate brain network, distinct from that which specifies the behavioral state of the subject.

## Conclusions

In summary, the occurrence of increasing network activity and improved learning performance that was most pronounced in individuals with low baseline performance and high disease burden is consistent with a compensatory response. At the region level, the orbitofrontal cortex may play a critical role in mediating this response. The inverse relationship between the network changes and disease burden suggests that this response is most relevant in pHD subjects nearing phenoconversion.
